# Unusual Presentation of Multisystemic Inflammatory Syndrome

**DOI:** 10.1155/2022/8442855

**Published:** 2022-08-10

**Authors:** Farah Alnoor Ebrahim, George Moturi, Newnex Mongare, Reena Shah

**Affiliations:** ^1^Department of Medicine, Faculty of Health Science, The Aga Khan University Hospital, Aga Khan University of Medical Collage of East Africa, Nairobi, Kenya; ^2^Department of Infectious Diseases, Faculty of Health Science, The Aga Khan University Hospital, Aga Khan University of Medical College of East Africa, Nairobi, Kenya

## Abstract

COVID-19 pneumonia in children presents with very mild symptoms through an entity of multisystem inflammatory syndrome and can result in a life-threatening hyperinflammatory condition, with involvement of at least four organ systems and a marked inflammatory state. We present an 18-year-old high school student who presented with a sore throat, macular rash, abdominal pain, diarrhea, fevers, and joint pains. He presented with acute kidney injury and confusion with multiple tests and was eventually diagnosed with multisystem inflammatory syndrome in children (MIS-C).

## 1. Case Presentation

An 18-year-old African male high school student with no prior comorbid conditions presented with a 2-day history of tonsillitis and was noted to have a macular rash that started on his chest and extended to his arm. He had received symptomatic treatment by a school clinician with oral chlorohexidine and lozenges. Three days into the initial symptom, he developed abdominal cramping and multiple episodes of non-bloody diarrhea, watery in nature, without nausea nor vomiting. He also had fevers and joint pains (both wrist, elbows, and knees). He was taken to a nearby health facility, where he was noted to have a high temperature and tachycardia. Within 24 hours in this facility, he developed confusion and was reported to have increasing creatinine, which rose from 200 *μ*mol/L to 400 *μ*mol/L. Treatment received included ceftriaxone 2 grams once a day for three days, paracetamol 1 g three times a day, loperamide 2 mg with every episode of diarrhea, and one dose of IV methylprednisolone 1 g. Given his deterioration, he was transferred to our facility for further evaluation and treatment.

On arrival at this hospital, he was tachycardic at 130 beats per minute, tachypneic with a respiratory rate of 50 breaths per minute, febrile at 40 degrees Celsius, hypotensive at 70/40 mmHg, and desaturating to 86% on room air. His GCS was 14/15; respiratory exam revealed bilaterally coarse crepitations throughout the lung fields with an audible S3 gallop rhythm on cardiovascular examination. Per abdomen was distended, tense with hypoactive bowel sounds. The musculoskeletal examination was unremarkable. He underwent acute management with fluid resuscitation, an urgent central line was placed, and he was initiated on inotropic support at 0.4 mcg per kilogram per minute.

The initial laboratory findings included a full hemogram which depicted an elevated white cell count of 27.12 × 10^9^/L, hemoglobin of 10.9 g/dl (normocytic normochromic), and platelets of 281 × 10^9^/L. Inflammatory markers were elevated with a procalcitonin of 71.58 ng/ml and C-reactive protein of 287 mg/L. The kidney fuctions were deranged with a creatinine of 494mmol/L, urea 26.70 mmlols/L, sodium 128 mmol/L, potassium 4.87mmol/L, and bicarbonate 15.5 mmol/L (blood gas analysis supoorted a metobaolic acisosis with a pH7.33, PCO2 23 PO2 122 and HCO3- 19.8). His liver function test represented ischemic hepatitis with an elevated AST of 1461 units/L ALT 812 units/L normal bilirubin (hepatitis screens A, B, and C were negative, including an HIV test). Urine analysis showed proteinuria with protein three-plus and blood four plus (with no red blood cells) and a urine albumin creatine ratio confirming proteinuria of 52.28 mg/mmol. The cardiac markers were elevated with a troponin of 17,137 ng/ml with an elevated creatine kinase of more than 7800 units/L. At this point, a diagnosis of rheumatic heart disease and rheumatic fever was entertained; an ASOT titer was obtained, being negative.

**Figure 1:**
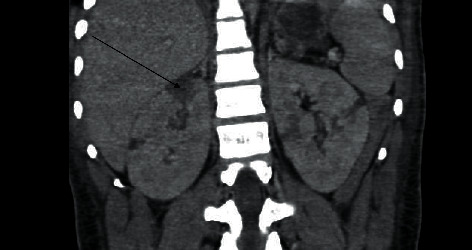
Computed tomography (CT) of the abdomen showing multiple hypodense wedge-shaped parenchymal defects involving the cortex and medulla consistent with renal infarcts (black arrow).

**Figure 2:**
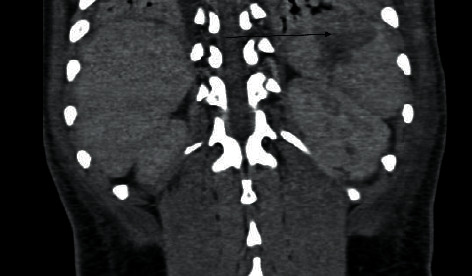
Computed tomography (CT) of the abdomen showing hypodense pyramidal wedge-shaped defect consistent with splenic infarcts (black arrow).

His radiological investigation included a chest X-ray that showed a feature of pulmonary edema and cardiomegaly; a 2D echo showed dilated cardiomyopathy with a low ejection fraction of 30 to 35% and normal valves. A CT scan abdomen showed distended bowel with splenic and renal infarcts as highlighted in Figures 1 and 2, and an HRCT redemonstrated the cardiomegaly and bi-basal lung atelectasis.

Multiple differential diagnoses were entertained at this point, such as vasculitis (antiphospholipid antibody syndrome, medium-sized vasculitis) and multisystemic inflammatory syndrome, which has been superimposed by septic shock. Within a few hours, the patient deteriorated, requiring intubation, and his norepinephrine requirements had increased to 0.7 mcg per kilogram per minute. He had been initiated on meropenem one gram three times a day and was started on urgent dialysis to help with the pulmonary edema, and supportive management was commenced for the fevers (given liver dysfunction, paracetamol was avoided, and other measures were integrated, which included cooling and icepacks). Because of the above differential diagnosis, an autoimmune panel was sent. Complement 3 and Complement 4 (C_3_ and C_4_) were low at 0.51 g/L and 0.04 g/L, respectively. The patient was initiated on a low-dose steroid pulse at 250 mg once a day and, due to the high risk of immunosuppression secondary to the steroids, was given broader cover with vancomycin 500 mg twice a day (renally adjusted doses); the rest of the autoimmune panel results are as follows: positive beta2-glycoprotein IgA antiphospholipid antibody and negative ANA, antiDS DNA, ENA, pANCA, and cANCA. COVID-19 PCR from tracheal aspirate and nasal swab was negative. However, to our surprise, the COVID-19 antibody test was positive, giving us strong evidence that our patient was suffering from the multisystemic inflammatory syndrome. By this point, all cultures, including blood and urine, had no growth.

**Figure 3:**
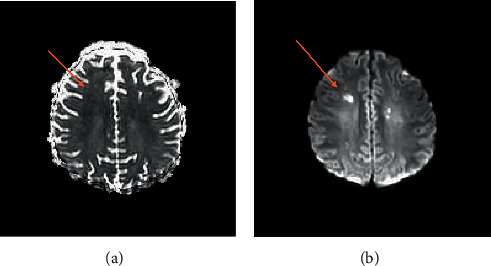
ADC mapping with an area of low signal intensity (left image orange arrow). Diffusion-weighted image showing high signal intensity (right image orange arrow).

**Figure 4:**
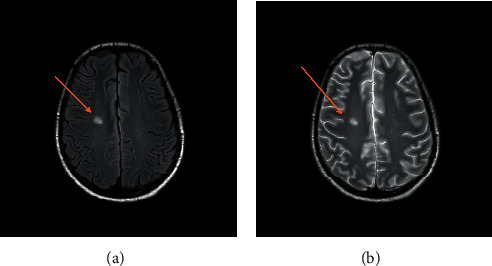
Flair MR image showing high signal intensity (left image orange arrow) and corresponding T2-weighted image showing an area of high signal intensity (right image orange arrow).

With the first pulse of steroids, the patient showed marked improvement with a decreasing pressor requirement from 0.7 mcgs of norepinephrine to 0.16 mcgs. Currently, the patient is doing well and has been successfully extubated, off inotropic support, with good urine output. He has remarkably overcome an acute process with four days of treatment with antibiotics and steroids and will continue on a tapering dose of steroids. He was successfully discharged from the hospital with some mild left leg weakness. As demonstrated by the MRI in Figures 3 and 4, the brain had developed small lacunar infarcts with residual neurological deficits.

## 2. Discussion

Multisystemic inflammatory syndrome in children (MIS-C) is where you have multiple organ inflammation, including the heart, lungs, kidneys, brain, skin, eyes, and gastrointestinal tract [1]. COVID-19 pneumonia in children presents very mild symptoms and can often be asymptomatic. There is a specific criterion that should be met for the diagnosis of MISC. As per the European and US center of disease prevention and control the diagnostic criteria is as follows: <21 years of age, fever for more than one day, laboratory evidence of inflammation, and more than two organ failure with a positive SARS-COVID 19 PCR or positive antigen test [2]. As seen in our case, the criteria were met given that the patient was 18 years old and had increased inflammatory markers evidenced by increased CRP and ferritin levels. The patient also had multiple organ failure including a positive antigen test, including the heart, lungs, kidneys, and brain.

The pathogenesis of COVID-19 pneumonia in children is similar to that of adults, where viral entry is via angiotensin-converting enzyme receptor 2, the difference being that fewer receptors in children give milder symptoms. In MIS-C, once the viral entry has been established, the early infection activates the macrophages after the T-helper cells causing cytokine release [3]. One after that develops a hyperimmune state by activating the B-cells causing the production of autoantibodies. The hyperimmune state leads to autoantibodies attacking the endothelial cell and gastrointestinal cells; myocardial damage's exact mechanism is unknown, but one can present with myocarditis or acute ischemic events [3].

The clinical presentation of MIS-C is a multisystemic disease, one of the most common manifestations is fevers occurring in 97–100% of patients, and dermatological manifestation includes rash and conjunctivitis (30–94%) [4]. Gastrointestinal (GI) manifestation are the most common presenting complaints which include abdominal pain, vomiting, and diarrhea [4]. Cardiac involvement includes features of myocarditis and ischemic events; around 31–100% of the patients may have left ventricular diastolic function evidenced by an echocardiogram [4]. Thrombotic complications like splenic infarction and cerebral strokes are also common manifestations [5]. On the other hand, renal infarctions are rare, and only a few cases reported in patients with COVID-19 pneumonia have developed renal infarctions [6–8]. Based on this evidence, one can see that the above patient presented with almost all the clinical signs making the diagnosis even stronger.

Acute kidney injury is prevalent amongst the SARS-COVID-19 patients. AKI and renal parenchymal disease may be caused by the ACE-2 receptors on the kidney parenchyma, causing microvascular injury triggered by the cytokine storm [9, 10]. As noted in outpatient, there was proteinuria, which has been reported in COVID-19 pneumonia patients. The theory is that viral replication occurs in the podocytes causing damage that can ensue in proteinuria [10]. Renal infarction resulting in AKI is rare; studies have shown renal infarction resulting in AKI but none requiring hemodialysis [7]. This case stands out as it is one of the only cases of renal infarction requiring renal replacement therapy. Most cases of renal infarction lead to AKI with no additional intervention required.

There is a unique coloration between SARS-COVID-19 pneumonia and antiphospholipid antibody syndrome; there can be an elevated anticardiolipin IgA antibody or an anti-B2-glycoprotein I IgA and IgG antibodies (as seen in our patient); this manifestation can lead to cerebral vascular infarctions, although studies do not support this hypothesis as in antiphospholipid syndrome it is the IgM and IgG titers that collate to thrombotic events [11]. Further studies need to be conducted to prove the thrombotic sequela amongst those with increased IgA titer, as clearly evidenced not only in our patient but in other case series conducted by Zhang et al., where all three patients had thrombotic events (multiple cerebral infarctions) and positive anticardiolipin IgA, anti-*β*2-glycoprotein I IgA, and IgG [12].

MIS-C and COVID-19 pneumonia is a pediatric syndrome characterized by increased inflammatory markers, fever, and multiorgan failure. There is a significant increase in the risk of morbidity and mortality, but some cases have a reasonable response rate, as seen in our patient. The unique events of positive IgA antiphospholipid antibodies and thrombotic events as seen in our patient may need further studies to prove any correlations.

## 3. Conclusion

COVID-19 pneumonia can lead to MIS-C resulting in multiple organ inflammation. Patients with MIS-C are more prone to thrombotic events and multiple organ failures. Therapies with steroids and intravenous immunoglobulins have been shown to improve outcomes significantly. AKI caused by renal infarction is rare; this may be the only case resulting in renal infarction requiring renal replacement therapy.
